# Hyperthermia Influences the Effects of Sodium Channel Blocking Drugs in Human-Induced Pluripotent Stem Cell-Derived Cardiomyocytes

**DOI:** 10.1371/journal.pone.0166143

**Published:** 2016-11-09

**Authors:** Ibrahim El-Battrawy, Siegfried Lang, Zhihan Zhao, Ibrahim Akin, Gökhan Yücel, Sophie Meister, Bence Patocskai, Michael Behnes, Boris Rudic, Erol Tülümen, Volker Liebe, Malte Tiburcy, Jennifer Dworacek, Wolfram-Hubertus Zimmermann, Jochen Utikal, Thomas Wieland, Martin Borggrefe, Xiao-Bo Zhou

**Affiliations:** 1 First Department of Medicine, Medical Faculty Mannheim, University of Heidelberg, Mannheim, Germany; 2 DZHK (German Center for Cardiovascular Research), Partner Sites, Heidelberg-Mannheim and Göttingen, Germany; 3 Institute of Pharmacology and Toxicology, University of Göttingen, Göttingen, Germany; 4 Skin Cancer Unit, German Cancer Research Center (DKFZ), Heidelberg and Department of Dermatology, Venereology and Allergology, University Medical Center Mannheim, University of Heidelberg, Mannheim, Germany; 5 Institute of Experimental and Clinical Pharmacology and Toxicology, Medical Faculty Mannheim, University of Heidelberg, Mannheim, Germany; 6 Institute of Cardiovascular Research, Southwest Medical University, Luzhou, Sichuan, China; Newcastle University, AUSTRALIA

## Abstract

**Introduction:**

Fever can increase the susceptibility to supraventricular and ventricular arrhythmias, in which sodium channel dysfunction has been implicated. Whether fever influences the efficacy of sodium channel blocking drugs is unknown. The current study was designed to investigate the temperature dependent effects of distinct sodium channel blocking drugs on the sodium currents in human induced pluripotent stem cell-derived cardiomyocytes (hiPSC-CMs).

**Methods and Results:**

hiPSC-CMs were generated from human skin fibroblasts of a healthy donor. The peak and late sodium currents (I_Na_), steady-state activation, inactivation and recovery from inactivation of I_Na_ in hiPSC-CMs were analyzed using the whole-cell patch clamp technique. The effects of different concentrations of the antiarrhythmic drugs flecainide, lidocaine, ajmaline and the antianginal drug ranolazine on I_Na_ were tested at 36°C and 40°C. Increasing the temperature of the bath solution from 36°C to 40°C enhanced the inhibition of peak I_Na_ but reduced the inhibition of late I_Na_ by flecainide and lidocaine. By contrast, increasing the temperature reduced the effect of ajmaline and ranolazine on the peak I_Na_ but not late I_Na_. None of the tested drugs showed temperature-dependent effects on the steady-state activation and inactivation as well as on the recovery from inactivation of I_Na_ in hiPSC-CMs.

**Conclusions:**

Temperature variation from the physiological to the febrile range apparently influences the effects of sodium channel blockers on the sodium currents. This may influence their antiarrhythmic efficacy in patients suffering from fever.

## Introduction

Ventricular tachyarrhythmias are the main reason for sudden cardiac death. Fever has been reported to be a trigger of ventricular tachyarrhythmias in patients with the Brugada syndrome (BrS) [1, 2, 3] and type 2 long QT syndrome [4, 5] but also in healthy individuals [6, 7]. BrS is characterized by coved type ST elevation in the precordial leads with incomplete or complete right bundle branch block and an increased risk for life-threatening ventricular tachyarrhythmias [8, 9]. Mutations in SCN5A, a gene encoding the cardiac sodium channel, have been linked to BrS [10]. The mutations may alter channel availability or gating, which lead to dysfunction of sodium channels in cardiomyocytes. Fever can influence both the availability and gating and thereby aggravate the dysfunction of these mutated channels and induce tachyarrhythmias in patients with BrS. Similarly, sodium blocking drugs like flecainide, ajmaline, disopyramide, procainamide, and lidocaine can also provoke the typical ECG changes and ventricular tachyarrhythmias in BrS by suppressing I_Na_ [11, 12, 13]. In individuals without genetic heart disease either fever or sodium channel blocking agents can provoke tachyarrhythmias, too. Therefore, it appears that both fever and sodium channel blockers can trigger tachyarrhythmias irrespective of ion channel mutations. However, whether fever influences the effect of the sodium channel blocking drugs is unknown. Some class I antiarrhythmic drugs are effective and clinically used for treatment of atrial and ventricular tachyarrhythmias. Therefore it is clinically relevant to investigate whether their efficacy in patients is altered by hyperthermia.

Since the successful reprogramming of adult somatic cells to induced pluripotent stem (iPS) cells and generation of functional cardiomyocytes from human iPS cells (hiPSC-CM) [14, 15, 16, 17], hiPSC-CMs have been demonstrated to have the electrophysiological and pharmacological properties including action potentials and responses to antiarrhythmic drugs which are similar to those of native cardiomyocytes [17, 18, 19]. hiPSC-CMs have also important advantages over heterologous expression systems like Xenopus oocytes, human embryonic kidney (HEK) cells and Chinese Hamster Ovary (CHO) cells lacking important constituents of cardiac ion channel macromolecular complexes that might be necessary for the normal electrophysiological characteristics, and transgenic animals possessing cardiac electrophysiological properties crucially different from that in humans. In addition, emerging evidences indicate that the hiPSC-CMs derived from patients with genetic heart diseases recapitulated the phenotype of the disease [5, 20, 21, 22, 23]. Thus, taking into account the hurdle for obtaining human ventricular cardiomyocytes, hiPSC-CMs provide an alternative tool for cardiovascular research. In this study we used therefore hiPSC-CMs to investigate the influence of hyperthermia on the effects of sodium channel blocking drugs on the sodium channel currents.

## Material and Methods

### Ethics statement

The skin biopsy from a healthy donor was obtained with written informed consent. The study was approved by the Ethics Committee of Medical Faculty Mannheim, Heidelberg University (approval number: 2009-350N-MA) and conducted in accordance with the Helsinki Declaration of 1975, as revised in 1983.

### Generation of human iPS cells

The human iPS cells (hiPSCs) were generated from primary human fibroblasts derived from skin biopsies of a healthy donor using lentiviral particles carrying the transactivator rtTA and an inducible polycistronic cassette containing the reprogramming factors OCT4, SOX2, KLF4 and c-MYC as previously described [24, 25]. Generated hiPSCs were cultured under feeder free conditions. To investigate pluripotency, hiPSCs were subjected to a teratoma-formation assay [24].

### Generation of hiPSC-CMs

Frozen aliquots of hiPSCs were thawed and cultured without feeder cells and differentiated into hiPSC-CMs as described with some modifications [26]. Culture dishes and wells were coated with Matrigel (Corning). Culture medium of hiPSCs was TeSR-E8 (Stemcell Technologies) and for hiPSC-CMs we used RPMI 1640 Glutamax (Life Technologies) containing sodium pyruvate, Penicillin / Streptomycin, B27 (Life Technologies) and ascorbic acid (Sigma Aldrich). Adding of CHIR99021 (Stemgent), BMP-4 (R&D Systems), Activin A (R&D Systems), FGF-2 (Miltenyi Biotec) and IWP-4 (Stemgent) at different time points induced the cells to differentiate into hiPSC-CMs during 3 weeks. During the third week a lactate (Sigma, Germany) containing RPMI-medium without glucose and glutamine (WKS, Germany) was added to select for cardiomyocyte cells. At 30 to 60 days of culture with basic culture medium, cardiomyocytes were dissociated from 24 well plates and plated on matrigel-coated 3.5 cm petri dishes for patch-clamp measurements: The cells were incubated with 300μl (150 U) collagenase CLS I (Worthington, Germany) for 40 min at 37°C, washed with PBS and incubated with 0.05% Trypsin-EDTA (Life Technologies) for 4 min at 37°C. After adding of RPMI medium containing 10% FCS, cells were centrifuged at 250 x g for 2 min at room temperature, the supernatant discarded and the cells resuspended with basic culture medium. The cells were plated on the 3.5 cm petri dishes at a density of 2–4 x 10^4^ cells/dish for subsequent patch-clamp experiments.

### Polymerase-Chain-Reaction Assays

For quantitative evaluation of the steady-state mRNA expression in hiPSC-CM cultures, total RNA was prepared using the RNeasy mini kit (Qiagen, Hilden, Germany), including DNAse treatment. Three micrograms of RNA were reverse transcribed and converted to cDNA with oligo(dT)_15_ primers using AMV reverse transcriptase according to standard protocols (Roche Applied Science, Germany). The cDNA was amplified by qPCR on a Stratagene MX 3005P real time cycler (Stratagene, USA) using a PCR-mix with hot start Taq DNA polymerase and SYBR-Green (Sibir Rox Hot Mastermix, Bioron, Germany) in the presence of sense and antisense primers (400 nM each). The sense- and antisense-primers for all human genes were supplied as RT^2^; qPCR Primer Assays from Qiagen (Germany). The PCR condition consisted of 95°C for 5 min (denaturation of DNA and activation of Taq polymerase), followed by 40 cycles of 95°C for 15 sec and 60°C for 1 min (annealing and extension), followed by melting-curve analysis to verify the correctness of the amplicon. Relative quantification of mRNA expression was calculated as follows: The expression of the mRNA of the gene of interest relative to the housekeeping gene GAPDH in samples from treated or untreated (Control) cells was calculated by the ΔΔCT method, based on the threshold cycle (CT), as fold change = 2^−Δ(ΔCT)^, where ΔCT = CT_gene of interest_ − CT_GAPDH_ and Δ(ΔCT) = ΔCT_treated_ −ΔCT _Control_ [27]. The data were acquired from two separate cell differentiation experiments (two biological replicates). From each cell differentiation two different cell culture wells were measured (totally four technical replicates).

### Patch-clamp

Standard patch-clamp recording techniques were used to measure the peak and late I_Na_ in the whole-cell configuration. Patch electrodes were pulled from borosilicate glass capillaries (MTW 150F; world Precision Instruments, Inc., Sarasota, FL) using a DMZ-Universal Puller (Zeitz-Instrumente Vertriebs GmbH, Martinsried, Germany) and filled with pre-filtered pipette solution (see below). Pipette resistance ranged from 1–2 MΩ. Electrode offset potentials were zero-adjusted before a Giga-seal was formed. After a Giga-seal was obtained, fast capacitance was first compensated and then the membrane under the pipette tip was disrupted by negative pressure to establish the whole-cell configuration. To determine the cell capacitance, a voltage pulse from -80 to -85 was given to record the cell capacitance transient current. Then we integrated the area under transient current curve and divide the area value by 5 mV to get the whole cell capacitance in pF. Thereafter the membrane capacitance (Cm) and series resistance (Rs) were compensated (60–80%). Liquid junction potentials were not corrected. Signals were acquired at 10 kHz and filtered at 2 kHz with the Axon 200B amplifier and Digidata 1440A digitizer hardware as well as pClamp 10.2 software (Molecular Devices, Sunnyvale, CA). The holding potential, if not otherwise indicated, was set at -80 mV to partially inactivate Na channels and reduce the peak I_Na_ to a proper range for adaquate voltage control of the currents.I_Na_ was elicited by using depolarizing pulses from -80 mV to different potentials. Measured currents were normalized to the membrane capacitance. Current-voltage (I-V) relationships were generated by plotting the current density against voltages from −120 to +20 mV (0.5 Hz). For assessing the activation and inactivation of peak I_Na_, the membrane conductance or relative current (I_m_/I_max_) were plotted against voltages from -120 to -10 mV and then fitted with Boltzmann distribution (f = Y_min_+(Y_max_-Y_min_)/(1+exp((x-x_0_)/b)), where Y_min_ and Y_max_ are the minimal and maximal values of currents, x is voltages and x_0_ is the voltage at 50% of activation or inactivation currents, and b is the slope factor.) to obtain the half maximal voltage (V0.5) of activation or inactivation. The membrane conductance (G_m_) was calculated as G_m_ = I/(E_m_− E_rev_), where I is macroscopic current, E_m_ is the test membrane potential, and E_rev_ is the reversal potential for sodium. To measure the recovery from inactivation of sodium channels, the protocol of double-pulse with increasing intervals was used. The peak I_Na_ elicited by the second pulse was normalized to that elicited by the first pulse and plotted against the intervals between the two pulses and then fitted with mono-exponential growth to get the time constant of recovery because mono-exponential function is sufficient for the best fitting of the values. Late I_Na_ was measured as the area under the current curve integrated from 50 to 350 ms after the beginning of the depolarization pulse. Recordings of whole I_Na_ at high temperature are technically challenging because of large amplitudes and fast gating kinetics. To optimize the measurements, in addition to using pipettes with low resistance, adjusting Cm and Rs to obtain minimal contribution of the capacitive transients, we used small cells with small I_Na_. Furthermore, to minimize the effects of run-down of I_Na_ on the results of experiments, we carefully monitored the time-dependent change of I_Na_. Recordings were started after the current reached a steady state, normally within 5 to 8 minutes with an amplitude <5 nA.

The bath solution contained (mmol/l): 50 NaCl, 110 CsCl, 1.8 CaCl_2_, 1 MgCl_2_, 10 Hepes, 10 glucose, 0.001 nifedipine, pH 7.4 (CsOH). Microelectrodes were filled with (mmol/l): 10 NaCl, 135 CsCl, 2 CaCl_2_, 3 MgATP, 2 TEA-Cl, 5 EGTA and 10 HEPES (pH7.2 CsOH). The concentration of NaCl was reduced to 20 mM in recordings of I_Na_ at a holding potential of -120 mV. Drugs were diluted from stock solution to extracellular solution in different concentrations. To assess the drug effect, stable I_Na_ was recorded before and after changing the extracellular solution containing a drug. Exchange of the drug-containing solutions was carried out sequentially from low to high concentrations by an 8-channel perfusion system OCTAFLOW-IITM (ALA Scientific Instruments Inc, Farmingdale, NY). The temperature of the perfused solution was maintained at 36°C or 40°C by the TC-20 npi temperature control system (npi electronic GmbH, Tamm, Germany).

Action potentials (APs) were recorded at 36°C. The filtering and sampling frequencies are the same as in current recordings (2 and 10 kHz). AP parameters (S1A Fig) were analyzed with an ISO-3 multitasking patch-clamp program (MFK M. Friedrich, Niedernhausen, Germany). In recordings of APs, the bath solution contains 130 mM NaCl, 5.9 mM KCl, 2.4 mM CaCl_2_, 1.2 mM MgCl_2_, 11mM glucose, 10 mM HEPES, pH 7.4 (NaOH). The pipette solution contains 10 mM HEPES, 126 mM KCl, 6 mM NaCl, 1.2 mM MgCl_2_, 5 mM EGTA, 11 mM glucose and 1 mM MgATP, pH 7.4 (KOH). The free Ca^2+^ concentration was adjusted to 0.1 μM by adding the appropriate amount of CaCl_2_ determined by aprogram for calculation of free Ca^2+^ concentration (Maxchelator).

### Drugs

Lidocaine (Sigma Aldrich) was dissolved in ethanol at a stock concentration of 300 mM. Flecainide acetate salt and ranolazine (Sigma Aldrich) were dissolved in DMSO to give a stock concentration of 100 mM. Ajmaline (MP Biomedicals) was dissolved in DMSO at a stock concentration of 30 mM.

### Statistical Analysis

If not otherwise indicated data are shown as mean ± SEM and were analyzed using InStat (GraphPad, San Diego, USA). By analyzing the data with the Kolmogorov Smirnov test it was decided whether parametric or non-parametric tests were used for analysis. For parametric data multiple comparisons with one-way ANOVA and Dunnett’s post-test were performed. For non-parametric data the Kruskal-Wallis test with Dunn’s multiple comparisons post-test was used. For repeated measurements, e.g. analysis of current-voltage relationships, parametric one-way repeated measures ANOVA with Dunnett’s multiple comparisons post-test was applied. Unpaired Student's t-test was used for comparisons of two independent groups with normal distribution. *p*<0.05 (two-tailed) was considered significant.

## Results

### Characterizations of hiPSC-CMs

**Fig 1:**
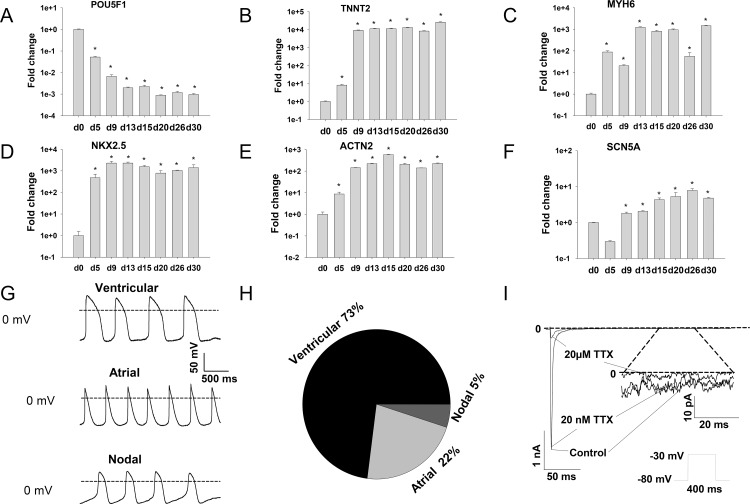
Characterizations of hiPSC-CMs. qPCR analysis was carried out to assess the expression of the pluripotency gene POU5F (A) and the cardiac genes TNNT2 (B), MYH6 (C), NKX2.5 (D) and ACTN2 (E) as well as the sodium channel gene SCN5A (F) at different times after onset of differentiation. Spontaneous APs were recorded in current-clamp mode without injection of current. Three forms of AP were observed (G-H). (I) Representative cardiac I_Na_, resistant to 20 nM TTX but sensitive to 20 μM TTX, was measured with the protocol shown as an inset at the bottom. The late I_Na_ was shown from 200 ms to 250 ms (indicated by dotted line) with different scale (inset). Values given are mean ± SEM. n = 4/2 (technical replicates/biological replicates). *, p<0.05 vs. d0.

To examine the gene expression patterns in hiPSCs and hiPSC-CMs, quantitative qPCR analysis was carried out at the beginning (day 0) and at different time points after onset of differentiation. The results showed that the pluripotency gene POU5F1 (POU class 5 homeobox 1, RefSeq NM_002701.5) decreased, while the typical cardiac genes, TNNT2 (Troponin T type 2, cardiac, RefSeq NM_000364), MYH6 (Myosin heavy chain 6, cardiac muscle, alpha, RefSeq NM_002471.3), NKX2.5 (NK2 homeobox 5, RefSeq NM_004387), and ACTN2 (Actinin, alpha 2, RefSeq NM_001103.3) increased over time, and reached a stable expression around two weeks after onset of differentiation (Fig 1A–1E). In addition, the expression of SCN5A (sodium channel, voltage-gated, type V, alpha subunit, RefSeq NM_000335.4), a gene encoding the sodium channel in the heart, started to increase on day 9 and reached a relatively stable level after day 15 (Fig 1F).

We further assessed the electrophysiological properties of the hiPS-CMs by measuring the action potential (AP) and membrane currents in the cells at 30 to 40 days after onset of differentiation. In current-clamp recordings, 73% (30/41) of the spontaneously beating cells showed ventricular-like, 22% (9/41) of the cells showed atrial-like and 5% (2/41) of the cells showed nodal-like APs (Fig 1G and 1H). The classification of APs into the three forms is based on AP shape, APD90 to APD50 (action potential duration at 90% to 50% repolarization) ratio, and maximum diastolic potential [22, 28]. The ventricular-like cell is characterized by an AP with significant plateau phase (APD90/50 ratio <1.4), the atrial-like cell shows a triangular-shaped AP (APD90/50 ratio >1.7), and the nodal-like cell (APD90/50 ratio between 1.4 and 1.7) displays a relatively depolarized diastolic potential and smaller amplitude AP (Fig 1G, S1 Table). In voltage-clamp recordings a large inward I_Na_, which was resistant to 20 nM but sensitive to 20 μM tetrodotoxin (TTX) (Figs 1I and S1B), was detected in all spontaneously contracted cells, indicating the expression of cardiac sodium channels in hiPSC-CMs.

### Effect of temperature variation on I_Na_

**Fig 2:**
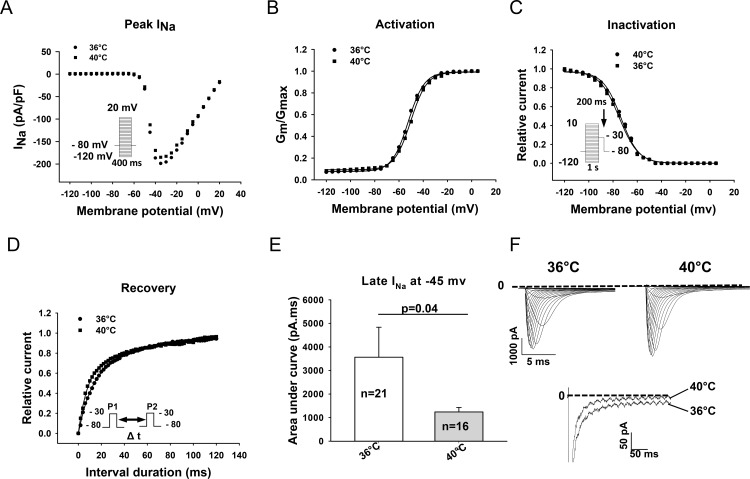
Effects of temperature variation on sodium channel currents and current kinetics. (A) Representative current-voltage relationships for peak I_Na_ acquired with indicated voltage protocols (the inset on the left) at 36°C and 40°C. (B) Normalized membrane conductance (G_m_/G_max_) versus membrane potentials demonstrating the steady-state activation of I_Na_ measured at 36°C and 40°C. (C) Normalized peak I_Na_ (I_m_/I_max_) measured at -30 mV with the indicated protocol (inset) versus membrane potentials demonstrating the steady-state inactivation of I_Na_ measured at 36°C and 40°C. (D) Recovery of I_Na_ from inactivation measured with the indicated protocol (inset) at 36°C and 40°C. (E) Late I_Na_ illustrated as the area under the current curve (pA*ms) between 50 and 350 ms of the pulse at -45 mV. (F) Examples of Na^+^ current traces showing the peak I_Na_ and late I_Na_ (at -40 mV) recorded at 36°C and 40°C. Values given are mean ± SEM. n, number of cells. ns, P>0.05.

To evaluate the effect of temperature variation on I_Na_, stable whole-cell I_Na_ was recorded using the same protocols at 36°C and 40°C (Fig 2). The current-density of peak I_Na_ was not significantly different at 36°C (271.4±41.4 pA/pF,n = 34) and at 40°C (207.2±27.8 pA/pF, n = 41, p>0.05) (Fi. 2A and F, S2 Table). However, the late I_Na_ was significantly reduced by 64% by increasing the temperature from 36°C to 40°C (Fig 2E and 2F). The areas under the current curves from 50 ms to 350 ms of the beginning of the depolarizing pulse, which were validated for late I_Na_ [29], were 3563.9±1274.4 ms*pA at 36° (n = 21) and 1237.4±191.7 ms*pA at 40°C (n = 16), respectively (P<0.05). We further assessed the influence of temperature on the steady-state activation, inactivation and the recovery from inactivation of I_Na_. At 36°C (n = 21) and 40°C (n = 16), the half-maximal activation potentials (-47.8±2.8 mV versus -50.0±3.5 mV), half-maximal inactivation potentials (-74.0±1.2 mV versus -72.3±1.0 mV) and time constants of recovery from inactivation (17.0±2.4 ms versus 21.1±4.3 ms) were not significantly different (Fig 2B–2D).

### Effect of sodium channel blocking drugs on peak I_Na_ at 36°C and 40°C

**Fig 3:**
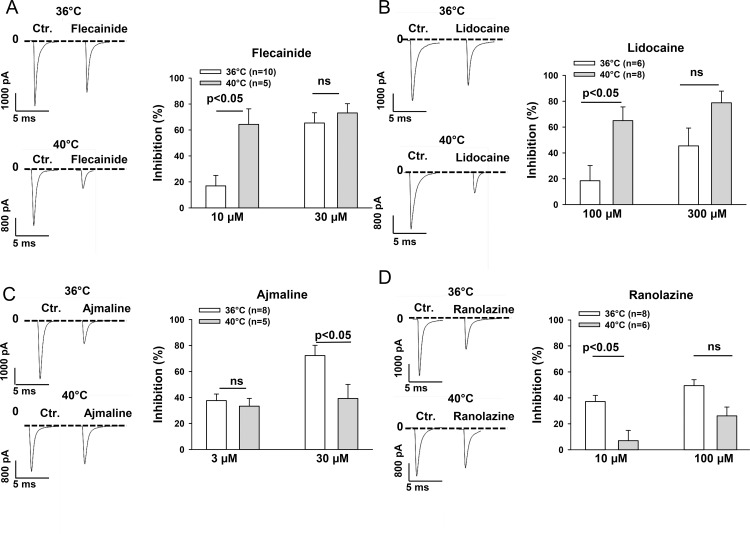
Hyperthermia enhanced the effect of flecainide and lidocaine but reduced the effect of ajmaline and ranolazine on the peak I_Na_. I_Na_ was recorded with the same protocol as that shown in Fig 1A in absence and presence of different concentrations of the tested drugs at 36°C and 40°C. Representative traces (left panels) and mean values (right panels) of percent inhibition of peak I_Na_ recorded at -35 mv in the presence of flecainide (A), lidocaine (B), ajmaline (C) and ranolazine (D) at 36°C and 40°C are shown by the bar graphs. Values given are mean ± SEM. n, number of cells. ns, P>0.05.

To check whether febrile temperature influences the effects of sodium channel blocking drugs flecainide, lidocaine, ajmaline and ranolazine on the peak I_Na_, the responses of the currents to these drugs in different concentrations were tested at 36°C and 40°C (Fig 3). All the four drugs inhibited I_Na_ in a concentration-dependent manner. The inhibition of the current by flecainide was enhanced by increasing temperature from 36°C to 40°C (Fig 3A). The statistical data from the current-voltage (I-V) curves at -35 mV demonstrated that the inhibition of the peak I_Na_ by 10 μM flecainide was significantly larger at 40°C (64.3±12.0% inhibition) than at 36°C (16.9±8.2% inhibition, P<0.05). Similarly, the significant effect of the temperature variation on efficacy of lidocaine was detected at 100 μM. The peak I_Na_ was more inhibited by 100 μM lidocaine at 40°C (65.0±10.6% inhibition) than at 36°C (18.5±11.8% inhibition, P<0.05) (Fig 3B). In contrast, the effects of ajmaline and ranolazine on peak I_Na_ were diminished by increasing the temperature from 36°C to 40°C. Ajmaline (30 μM) and ranolazine (10 μM) showed a smaller effect on peak I_Na_ at 40°C (inhibition by ajmaline, 39.2±10.9%; inhibition by ranolazine, 7.0±7.9%) than at 36°C (inhibition by ajmaline, 72.3±7.9%; inhibition by ranolazine, 37.1±4.8%; p<0.05) (Fig 3C and 3D). Please note that the significant differences of drug effects between 36°C and 40°C were detected only in certain concentrations of drugs, indicating that the temperature-dependent effects were also concentration-dependent (Fig 3). To check whether the membrane potential influences the temperature-dependent effects, we repeated the experiments with 100 μM lidocaine and 10 μM ranolazine in cells with the holding potential of -120 mV. Similarly as in cells with the holding potential of -80 mV, hyperthermia enhanced the effect of lidocaine but reduced the effect of ranolazine (S2 Fig), suggesting that temperature-dependent effects of drugs may be voltage-independent.

### Effect of sodium channel blocking drugs on late I_Na_ at 36°C and 40°C

**Fig 4:**
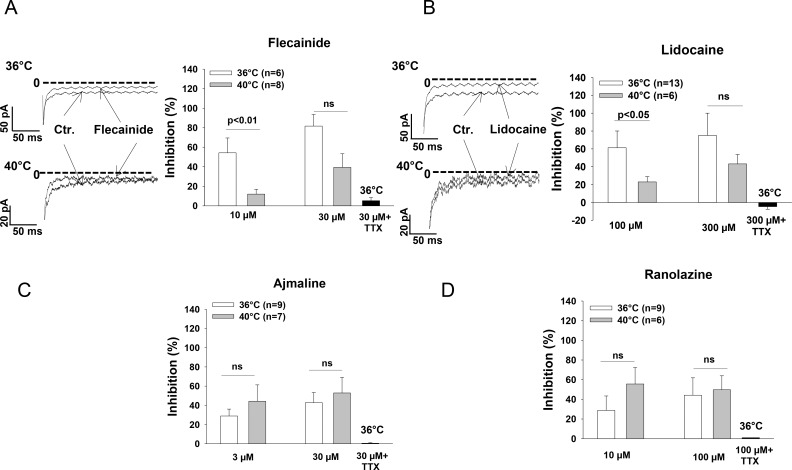
Hyperthermia diminished the effect of flecainide and lidocaine on the late I_Na_. The late I_Na_ was recorded by a depolarizing pulse from the holding potential of -100 mV to -45 mV with the duration of 400 ms. The area under the current curve was measured between 50 ms and 350 ms of the depolarizing pulse to illustrate the quantity of the late I_Na_. Mean values of the percent inhibition of late I_Na_ in the presence of flecainide (A), lidocaine (B), ajmaline (C) and ranolazine (D) at 36°C and 40°C as well as 36°C plus 20 μM TTX are shown by the bar graphs. Representative traces of late I_Na_ inhibited by flecainide and lidocaine at 36°C and 40°C are also shown. Values given are mean ± SEM. n, number of cells. ns, P>0.05.

Next, we assessed the effects of the tested drugs on the late I_Na_ at 36°C and 40°C. On the contrary to the effect on peak I_Na_, the inhibitory effect of flecainide and lidocaine on the late I_Na_ was reduced by increasing the temperature (Fig 4A and 4B). The inhibition of late I_Na_ by 10 μM flecainide and 100 μM lidocaine at 40°C was 12.0±5.0% and 23.0±5.9%, whereas the respective values at 36°C were 54.3±15.2% and 61.3±18.7% (p<0.05), respectively. However, the effect of ajmaline and ranolazine on the late I_Na_ was not statistically different at 36°C and 40°C (Fig 4C and 4D). In the presence of 20 μM TTX, all the four drugs lost their inhibitory effect (Figs 4 and S4), excluding the contribution of non-sodium channel currents to the observed inhibition of late INa.

### Effect of sodium channel blocking drugs on channel kinetics at 36°C and 40°C

**Fig 5:**
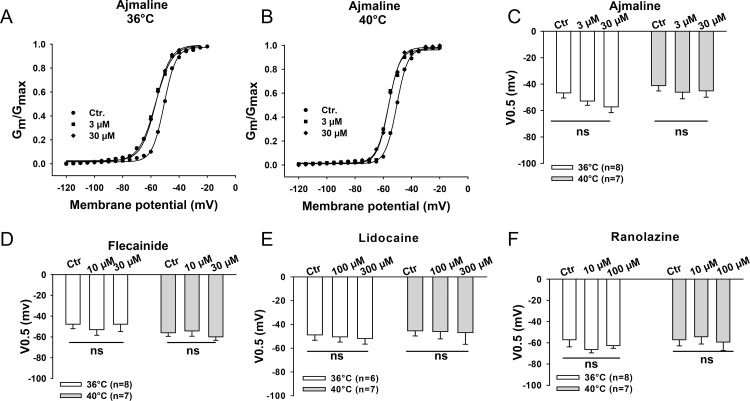
Effects of the Na channel blocking drugs on the activation of peak I_Na_. Peak I_Na_ was recorded with the same protocol as that shown in Fig 1A and converted to membrane conductance (G_m_) and normalized to the maximum (G_max_). The normalized G_m_ was plotted against the membrane potentials to obtain the activation curves. The curves are fitted by Boltzmann equation to obtain the half maximal voltage (V0.5) of activation. (A)-(B) Representative activation curves in the presence of different concentrations of ajmaline at 36°C and 40°C. Mean values of V0.5 of the activation curves in the presence of ajmaline (C), flecainide (D), lidocaine (E) and ranolazine (F) at 36°C and 40°C are shown by the bar graphs. Values given are mean ± SEM. n, number of cells. ns, P>0.05.

**Fig 6:**
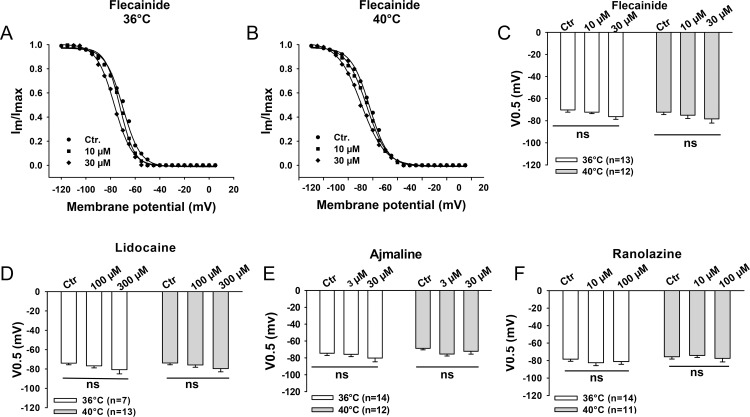
Effects of the Na channel blocking drugs on the inactivation of peak I_Na_. The normalized peak I_Na_ (I_m_/I_max_) was plotted against the membrane potentials to obtain the inactivation curves. The curves are fitted by Boltzmann equation to obtain the half maximal voltage (V0.5) of inactivation. (A)-(B) Representative inactivation curves in the presence of different concentrations of flecainide at 36°C and 40°C. Mean values of V0.5 of the inactivation curves in the presence of flecainide (C), lidocaine (D), ajmaline (E) and ranolazine (F) are shown by the bar graphs. Values given are mean ± SEM. n, number of cells. ns, P>0.05.

**Fig 7:**
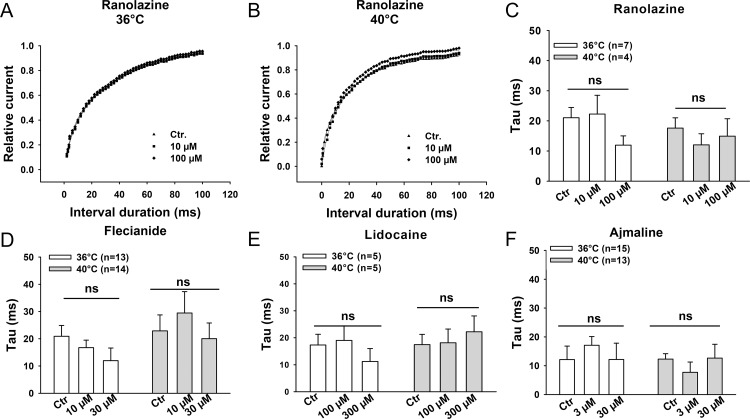
Effects of the Na channel blocking drugs on the recovery from inactivation of peak I_Na_. (A)-(B) Representative recovery curves in the presence of different concentrations of ranolazine at 36°C and 40°C. Mean values of the time constants of the recovery from inactivation of peak I_Na_ in the presence of ranolazine (C), flecainide (D), lidocaine (E) and ajmaline (F) are shown by the bar graphs. Values given are mean ± SEM. n, number of cells. ns, P>0.05.

Finally, we analyzed the effects of flecainide, lidocaine, ajmaline and ranolazine on the channel gating kinetics including the steady-state activation (Fig 5), steady-state inactivation (Fig 6) and recovery from the inactivation of peak I_Na_ (Fig 7) at 36°C and 40°C. None of the tested drugs showed temperature-dependent effects on these three channel gating parameters. We also analyzed the use-dependent blockade of I_Na_ by lidocaine and ranolazine (S3 Fig). The development of use-dependent blockade by lidocaine but not by ranolazine was accelerated by increasing the temperature to 40°C.

## Discussion

To our knowledge, this is the first study in hiPSC-CMs that reveals temperature-dependent effects of the sodium channel blocking drugs on I_Na_.

For this study we successfully generated hiPS-CMs with the majority (73%) of cells showing ventricular-like APs (S1 Table). The successful differentiation of hiPS cell into cardiomyocytes was confirmed by the following evidences: 1) mRNA expression of the pluripotency gene POU5F1 decreased (Fig 1A) during the differentiation, while the cardiac genes, TNNT2, MYH6, NKX2.5, and ACTN2 increased (confirmed by qPCR, Fig 1B–1E); 2) the cells started to beat spontaneously around 10 days after onset of differentiation (data not shown); 3) the spontaneously beating cells showed action potentials of cardiac morphology (Fig 1G and 1H); 4) the cells express functional cardiac sodium channels, which was confirmed by qPCR and patch clamp measurements (Figs 1F, 1I and S1B).

The cardiac sodium channels carry the predominat depolarizing inward current responsible for the generation of the rapid upstroke of the cardiac AP, and play a vital role in the conduction of excitation in working myocardium and the Purkinje conduction system. In addition, the inward I_Na_, especially the sustained late I_Na_, also contributes to the duration of AP (APD). It is well-known that the dysfunction of cardiac Na^+^ channels may increase susceptibility to tachyarrhythmias. Multiple factors including mutations in SCN5A gene, fever and Na^+^ channel blockers are related to arrhythmogenesis. However, whether those factors can influence each other when two or more of them act together on I_Na_ has not been well investigated. The correlation between the channel mutation and fever has been established in BrS [2, 30, 31, 32]. We report here that the temperature variation from the physiological to the febrile range influenced both I_Na_ and the effects of the sodium channel blocking drugs flecainide, lidocaine, ajmaline, and ranolazine on I_Na_ in hiPSC-CMs from a healthy donor. Increasing the temperature of the bath solution from 36°C to 40°C reduced significantly the late but not peak I_Na_ (Fig 2A, 2E and 2F). The reduction in late I_Na_ may result in shortening of APD and QT interval. This is consistent with a report that in healthy subjects the QT interval significantly shortened during fever [33]. Consistent with a previous study showing that peak I_Na_ was similar at 37°C and 42°C although enhanced from 22°C to 37°C [34], hyperthermia (40°C), in our study, did not significantly change the amplitude of peak I_Na_ (Fig 2A and 2F). However, hyperthermia enhanced the inhibition of peak I_Na_ by flecainide and lidocaine (Fig 3A and 3B) but reduced the inhibition of peak I_Na_ by ajmaline and ranolazine (Fig 3C and 3D). These data suggest that flecainide or lidocaine may produce a greater, whereas ajmaline and ranolazine may induce a smaller effect on the excitation spread in the heart of patients with fever compared with normal temperature. On the other hand, hyperthermia diminished the inhibitory effect of flecainide and lidocaine but not of ajmaline and ranolazine on the late I_Na_ (Fig 4). This suggests that the APD-shortening effect of lidocaine may be smaller in fever than in normal body temperature. Flecainide, ajmaline and ranolazine have been reported to block also potassium channels [35, 36, 37], and thus may prolong APD. Thereby, whether the effect of flecainide, ajmaline and ranolazine on APD is influenced by fever will be determined by their total effects on both the inward sodium and the outward potassium channel currents in vivo in febrile state. It was beyond the scope of this study to investigate the temperature-dependent effects of drugs on currents other than I_Na_. Another role played by the cardiac I_Na_, especially the late I_Na_, is to increase the intracellular Ca^2+^ level through the enhanced reverse Na^+^/Ca^2+^ exchange current. Increased intracellular Ca^2+^ can also affect the electrophysiological property through different mechanisms, including delayed afterdepolarization (DAD). An enhanced late I_Na_ is arrhythmogenic because it enhances triggered activity via DAD. Late I_Na_ Inhibitors, like ranolazine, may reduce or prevent the arrhythmogenic effects of late I_Na_. Our data show that the effect of ranolazine on late I_Na_ was similar at 36°C and 40°C, suggesting that fever has minor influence on the efficacy of ranolazine based on its late I_Na_ blocking actions. In addition, the temperature influence on the drug effects was concentration-dependent (Figs 3 and 4). This suggests that a significant influence of temperature on drug effects occurs only at certain concentrations of drugs. Therefore both temperature and drug dosage are important for the effects of sodium channel blocking drugs used in patients with fever.

To elucidate possible mechanisms underlying the influence of hyperthermia on the sodium channel blocking effects of the drugs, we analyzed the steady-state activation and inactivation as well as recovery from inactivation of the channel in presence of flecainide, lidocaine, ajmaline and ranolazine at 36°C and 40°C. The results displayed no significant temperature-dependent differences of the effects of all the four drugs on those three channel-gating parameters (Figs 5–7). This suggests that the temperature-dependent effects of the tested drugs are probably based on the alteration of the availability rather than the gating kinetics of sodium channels in the cell membrane, which is similar to the effect of isoproterenol on the lidocaine-induced suppression of I_Na_ [38], but inconsistent with the results of a study showing temperature dependence of sodium channel block by lidocaine [33]. In that study, the effect of lidocaine (50 μM) on blocking peak I_Na_ between 10°C and 25°C in Purkinje fibers was investigated and the temperature-dependence of channel gating kinetics in presence of lidocaine was observed. Furthermore, another study showed that recovery of I_Na_ blocked by *O*-demethyl encainide, a metabolite of encainide with potent antiarrhythmic effects, was drastically reduced by lower temperature between 15°C and 25°C [39]. Gating kinetics of the Na^+^ channel are known to be sensitive to temperature in the unphysiological range (<37°C) [34, 40]. It would be expected to detect the temperature-dependent effects of Na^+^ channel blockers on the channel gating kinetics at low temperature. Our data (S2 Table) and the previous study by *Keller* and co-workers [34] show that steady-state activation and inactivation as well as recovery from inactivation of normal Na^+^ channels were not significantly different at physiological versus febrile temperature. However, I_Na_ and the time constants of recovery from inactivation at room temperature are significantly different from that at physiological temperature (S2 Table). This suggests that the temperature-dependent change of Na^+^ channel gating kinetics happens mainly in a certain temperature range lower than body temperature. This may partially explain why we did not observe any difference of those three channel gating parameters in the presence of the tested drugs at 36°C versus 40°C.

It is well-known that the membrane potential is important for the effects of Na channel blocking antiarrythmic drugs. The drug affinity for the open (e.g. disopyramide) and inactivated (e.g. lidocaine) states is higher than that for the resting state of Na channels. At -80 mV, at which the aforementioned temperature-dependent effects were detected, more channels are in inactivated the state than at more hyperpolarized potentials, and the blockade by drugs will be enhanced. The temperature influence on drug effects may also vary with membrane potentials. Therefore we assessed the effects of lidocaine and ranolazine on I_Na_ recorded with a holding potential of -120 mV. The results showed that, as at -80 mV, hyperthermia enhanced lidocaine but reduced ranolazine effect on peak I_Na_ (S2 Fig), suggesting that the temperature-dependent effects be probably membrane potential independent.

Another important feature of class I antiarrythmic drugs is their use-dependent block. Na channels become blocked during depolarization and recovery from blockade occurs between depolarizations. If the recovery is not complete, accumulation of block occurs during consecutive depolarizations until a steady-state is reached in which the amount of block acquired during depolarization equals that recovered between depolarizations. If temperature influences either the block development or the recovery speed, it may change the blockade accumulation. Our data (S3 Fig) show that hyperthermia accelerated the block accumulation of I_Na_ by lidocaine but not by ranolazine. This suggests that hyperthermia either speeded up the association or slowed the dissociation of lidocaine, but either did change both the association and dissociation of ranolazine or changed them in the same direction. From these results we cannot explain opposite effects induced by hyperthermia on the steady-state block of I_Na_ by lidocaine and ranolazine, but we can see that hyperthermia can influence some features of different drugs diferentially. Besides the membrane potential drug features like charge, acid dissociation constant and lipophilicity are also important for association to and dissociation from ion channels. How hyperthermia influences the association and dissociation of flecainide, lidocaine, ajmaline and ranolazine, and the exact reason for the enhanced and reduced block at 40°C need to be further addressed. Flecainide is a class Ic antiarrhythmic agent used to prevent tachyarrhythmias such as atrial fibrillation [41]. Ajmaline, a class Ia antiarrhythmic drug, has been used as treatment of Wolf-Parkinson-White syndrome and ventricular tachycardia [42, 43]. Lidocaine, a class Ib antiarrhythmic drug, plays a central role as an anesthetic drug. It plays a minor role in clinical cardiology at the present time. Ranolazine is an antianginal agent with antiarrhythmic effect and used in patients with chronic stable angina [44]. At least for flecainide, lidocaine and ranolazine the concentrations at which we observed alterations are near the therapeutic range. Therefore our data suggest that fever may influence their sodium channel blocking effects and thus their efficacy in patients. In addition, suppression of I_Na_ by other drugs with sodium channel blocking effect, either as a therapeutic or a side effect, may be likewise influenced by fever.

## Study Limitations

Some limitations should be considered in extrapolating the data from the current study. hiPSC-CMs from one healthy donor were used for this study. hiPSC-CMs possess similarities but also distinct differences in their physiological properties when compared to adult human cardiomyocytes. hiPSC-CMs showed the APs of immature myocytes, e.g., spontaneous activity and lower maximal diastolic potentials (MDP). The reduced MDP may influence some ion channels and therefore impact their contributions to APs. This influence should be considered in interpretation of the experimental data from hiPSC-CMs. Another consideration that should be made is that the characters of hiPSC-CMs may vary with different gene reprogramming, cell culture and differentiation methods as well as ages (differentiation and culture time) of cells. Owing to limited availability of cells, human ventricular cardiomyocytes were not used for comparison in this study. Therefore we cannot rule out the differences among individuals and between hiPSC-CMs and adult human cardiomyocytes regarding the drug effects in different temperatures. In addition, the investigation was not performed in hiPSC-CMs from patients carrying SCN5A mutations. Some mutations in SCN5A channel are implicated in sodium channel dysfunction and fever-triggered tachyarrhythmias. Whether the effects of Na channel blocking drugs on wildtype or mutated channels are influenced by hyperthermia in the same manner remains to be addressed. Finally, the holding potential was set at -80 mV to partially inactivate Na channels and reduce the peak I_Na_ to a proper range for adequate voltage control of the currents. This potential is close to V0.5 of the inactivation which is at the steepest part of inactivation curve. Therefore small changes in either membrane potential or voltage dependency of the channel inactivation may have large effects on measured currents. The former changes the number of inactivated channels, the later changes the steepness or V0.5 of inactivation curve. This concern, however, can be avoided, if the peak I_Na_ is controlled in a proper rang either by choosing small cells with small I_Na_ or reducing the driving force for I_Na_ when the holding potential is kept away from V0.5 (< -100 mV). Nevertheless, the novel findings in this study may trigger further studies, either experimental or clinical.

## Conclusions

Our data demonstrated that hyperthermia influences the effects of sodium channel blocking drugs on cardiac sodium currents. This may influence their antiarrhythmic efficacy in patients suffering from fever.

## Supporting Information

S1 Fig Electrophysiological characterizations of hiPSC-CMs. **(A)** Schematic diagram of an action potential (AP) trace recorded by patch-clamp in a hiPSC-CM, showing how AP parameters were analyzed. RP, resting potential; Vmax, maximal depolarization velocity; APA, amplitude of AP; APD50 and APD90, the AP duration at 50 and 90% repolarization. (**B**) Averaged values of peak I_Na_ in absence (Ctr) and in presence of 20 nM and 20 μM TTX. n, cell number; ns, p>0.05 vs. Ctr; *, p<0.05 vs. Ctr.
(TIF)

S2 Fig Effects of lidocaine and ranolazine on peak I_Na_ at the holding potential of -120 mV. (A) Representaive traces of I_Na_ evoked by 100 ms pulses from -120 mV to -40 mV in absence (Ctr) and presence of 100 μM lidocaine at 36°C and 40°C. (B) Representaive traces of I_Na_ in absence (Ctr) and presence of 10 μM ranolazine at 36°C and 40°C. (C) Averaged values of per cent block of peak I_Na_ by lidocaine and ranolazine at the holding potential of -120 mV. n, cell number.
(TIF)

S3 Fig Use-dependent block of peak I_Na_ by lidocaine and ranolazine at 36°C and 40°C. From holding potential of -80 mV, 40 pulses with the duration of 100 ms were applied to -30 mV at intervals of 300 ms. Peak I_Na_ evoked by every pulse (I_pn_) was subtracted from that evoked by the first pulse (I_p1_) normalized to I_p1_ (I_p1_-I_pn_/I_first_). The normalized values of I_Na_ averaged from 6 cells were plotted as a function of pulse number to illustrate the development of use-dependent blockade by lidocaine (A) and ranolazine (B) at 36°C and 40°C.
(TIF)

S4 Fig TTX blocked I_Na_ and the effects of drugs on I_Na_. Shown are representaive I_Na_ traces recorded at -40 mV with the holding potential of -80 mV. 20 μM TTX inhibited both peak (A) and late (B) I_Na_, and also prevented the effects of 30 μM flecainide (C), 100 μM lidocaine (D), 30 μM ajmaline (E) and 100 μM ranolazine on late I_Na_.
(TIF)

S1 Table Action potential properties of hiPS-CMs paced at 1Hz. (DOCX)

S2 Table Properties of I_Na_-kinetics in iPS-CMs at 22°C, 36°C and 40°C. (DOC)
