# Supplementary figures and images for: Hyperthermia Influences the Effects of Sodium Channel Blocking Drugs in Human-Induced Pluripotent Stem Cell-Derived Cardiomyocytes
Source: PLoS One. 2016 Nov 9;11(11):e0166143. doi: 10.1371/journal.pone.0166143 (PMC5102382; doi:10.1371/journal.pone.0166143)

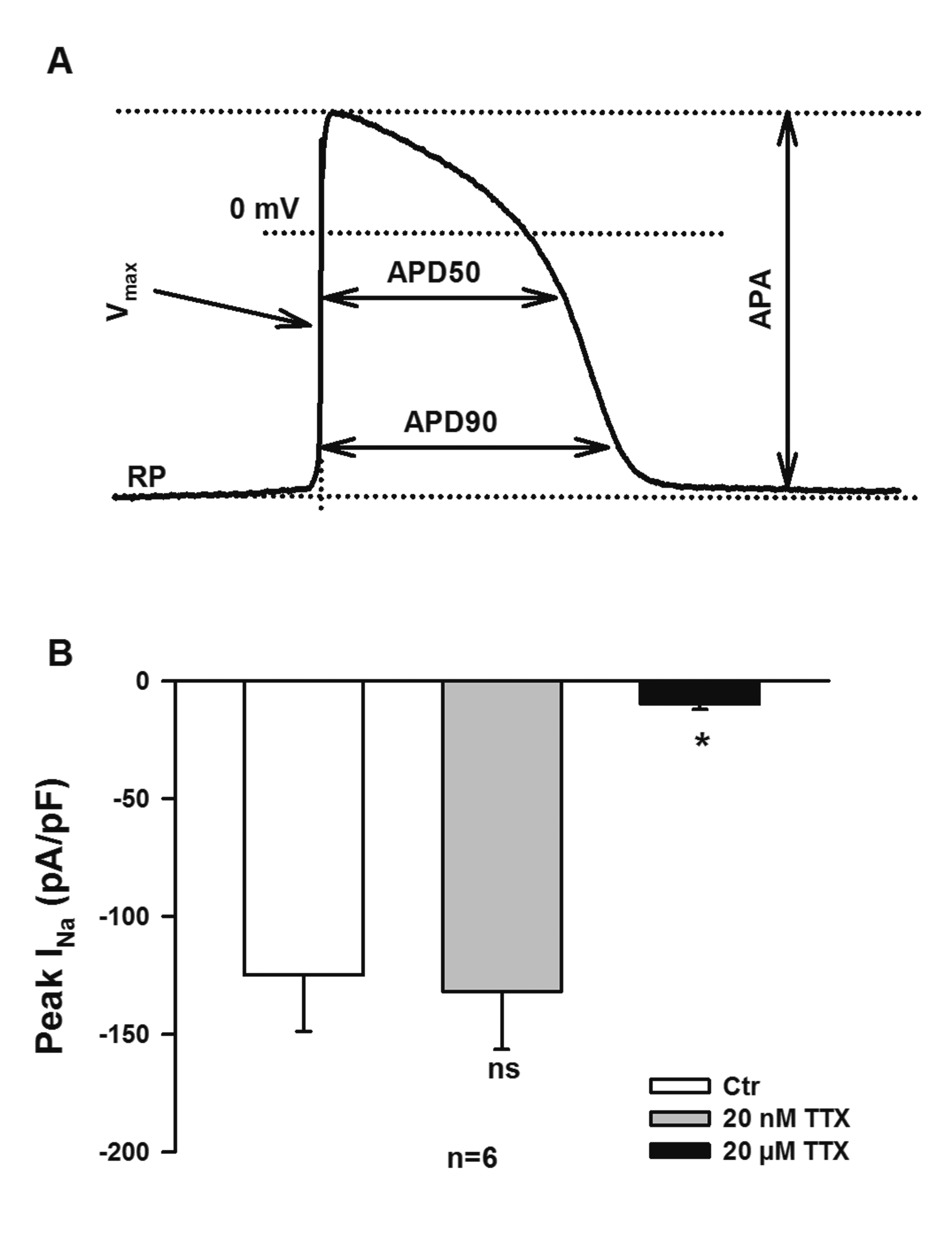

Supplement: S1 Fig — (A) Schematic diagram of an action potential (AP) trace recorded by patch-clamp in a hiPSC-CM, showing how AP parameters were analyzed. RP, resting potential; Vmax, maximal depolarization velocity; APA, amplitude of AP; APD50 and APD90, the AP duration at 50 and 90% repolarization. (B) Averaged values of peak INa in absence (Ctr) and in presence of 20 nM and 20 μM TTX. n, cell number; ns, p>0.05 vs. Ctr; *, p<0.05 vs. Ctr. (TIF) [file pone.0166143.s001.tif]

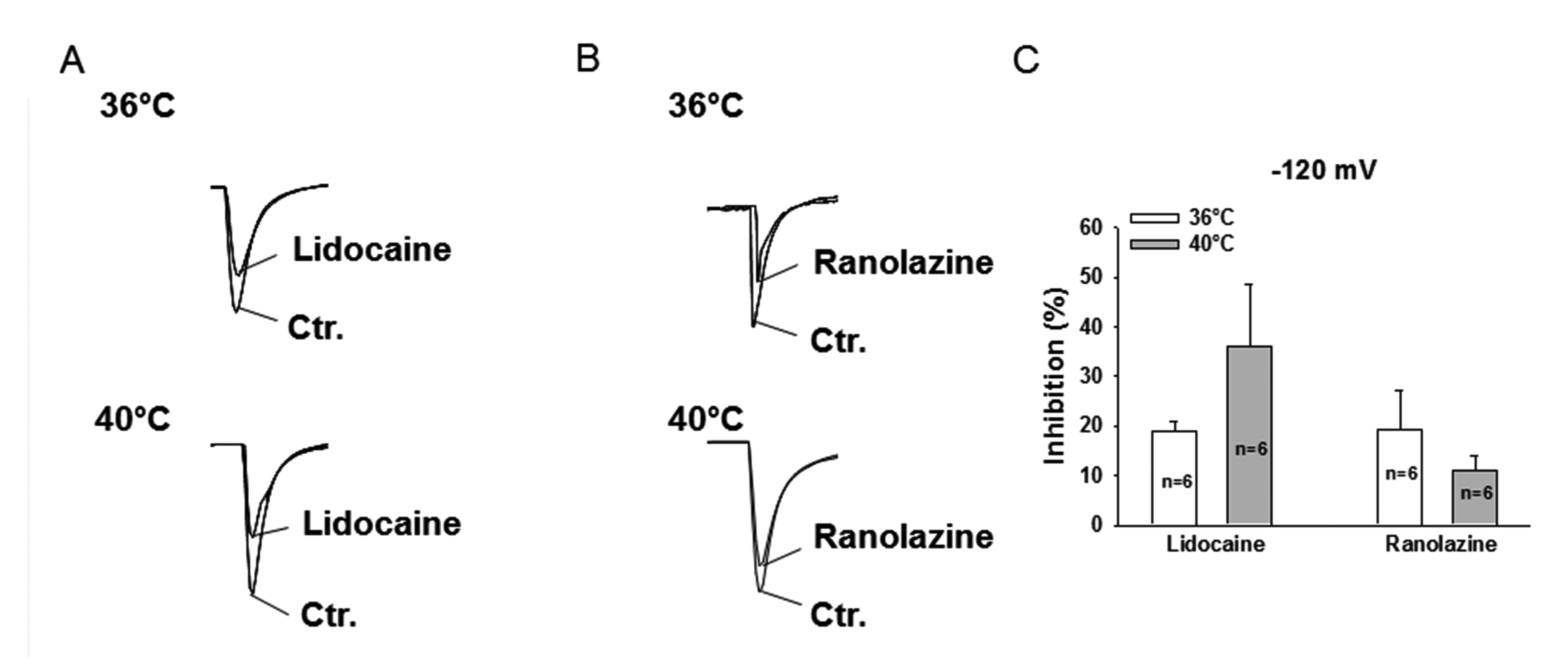

Supplement: S2 Fig — (A) Representaive traces of INa evoked by 100 ms pulses from -120 mV to -40 mV in absence (Ctr) and presence of 100 μM lidocaine at 36°C and 40°C. (B) Representaive traces of INa in absence (Ctr) and presence of 10 μM ranolazine at 36°C and 40°C. (C) Averaged values of per cent block of peak INa by lidocaine and ranolazine at the holding potential of -120 mV. n, cell number. (TIF) [file pone.0166143.s002.tif]

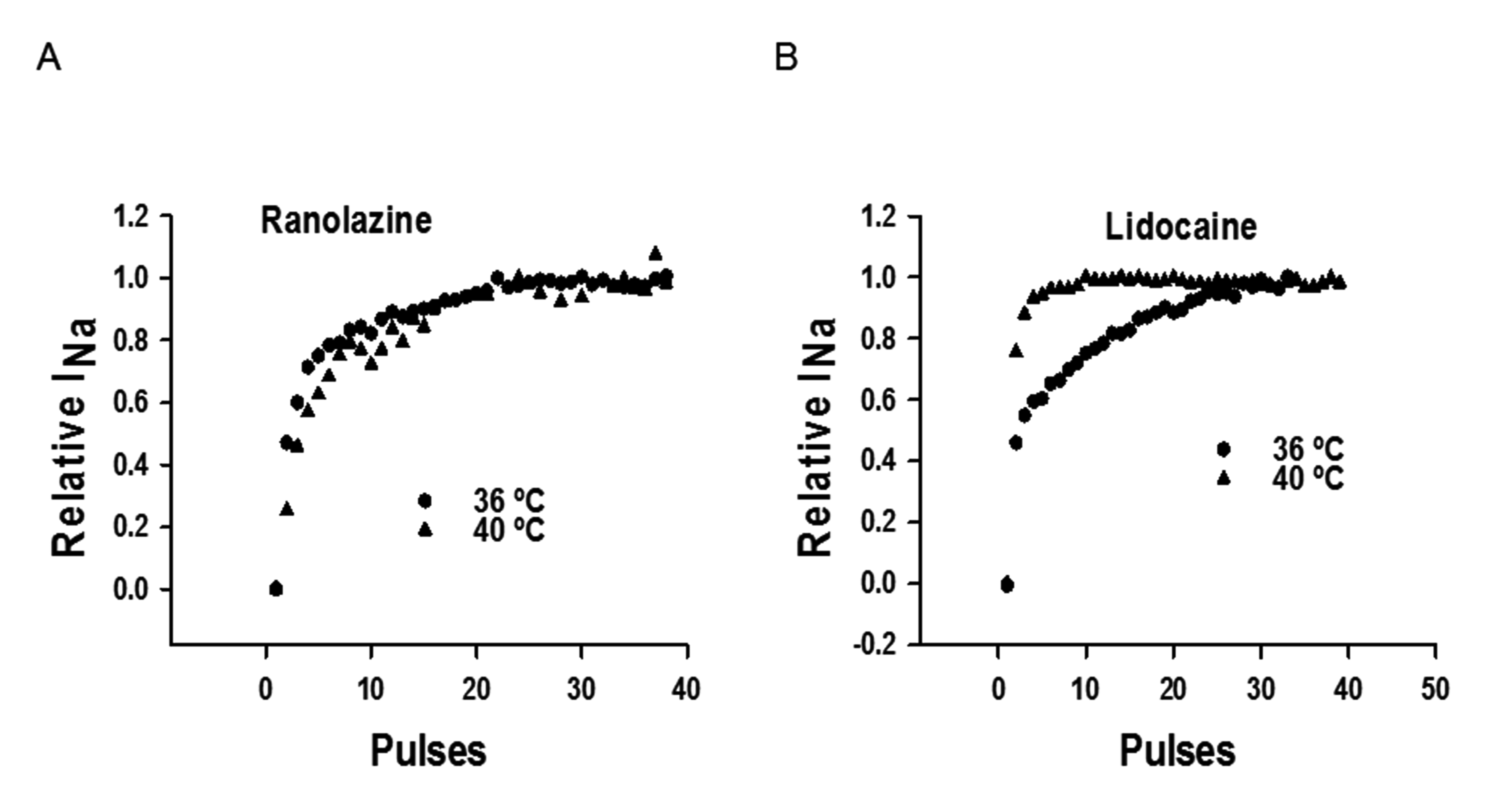

Supplement: S3 Fig — From holding potential of -80 mV, 40 pulses with the duration of 100 ms were applied to -30 mV at intervals of 300 ms. Peak INa evoked by every pulse (Ipn) was subtracted from that evoked by the first pulse (Ip1) normalized to Ip1 (Ip1-Ipn/Ifirst). The normalized values of INa averaged from 6 cells were plotted as a function of pulse number to illustrate the development of use-dependent blockade by lidocaine (A) and ranolazine (B) at 36°C and 40°C. (TIF) [file pone.0166143.s003.tif]

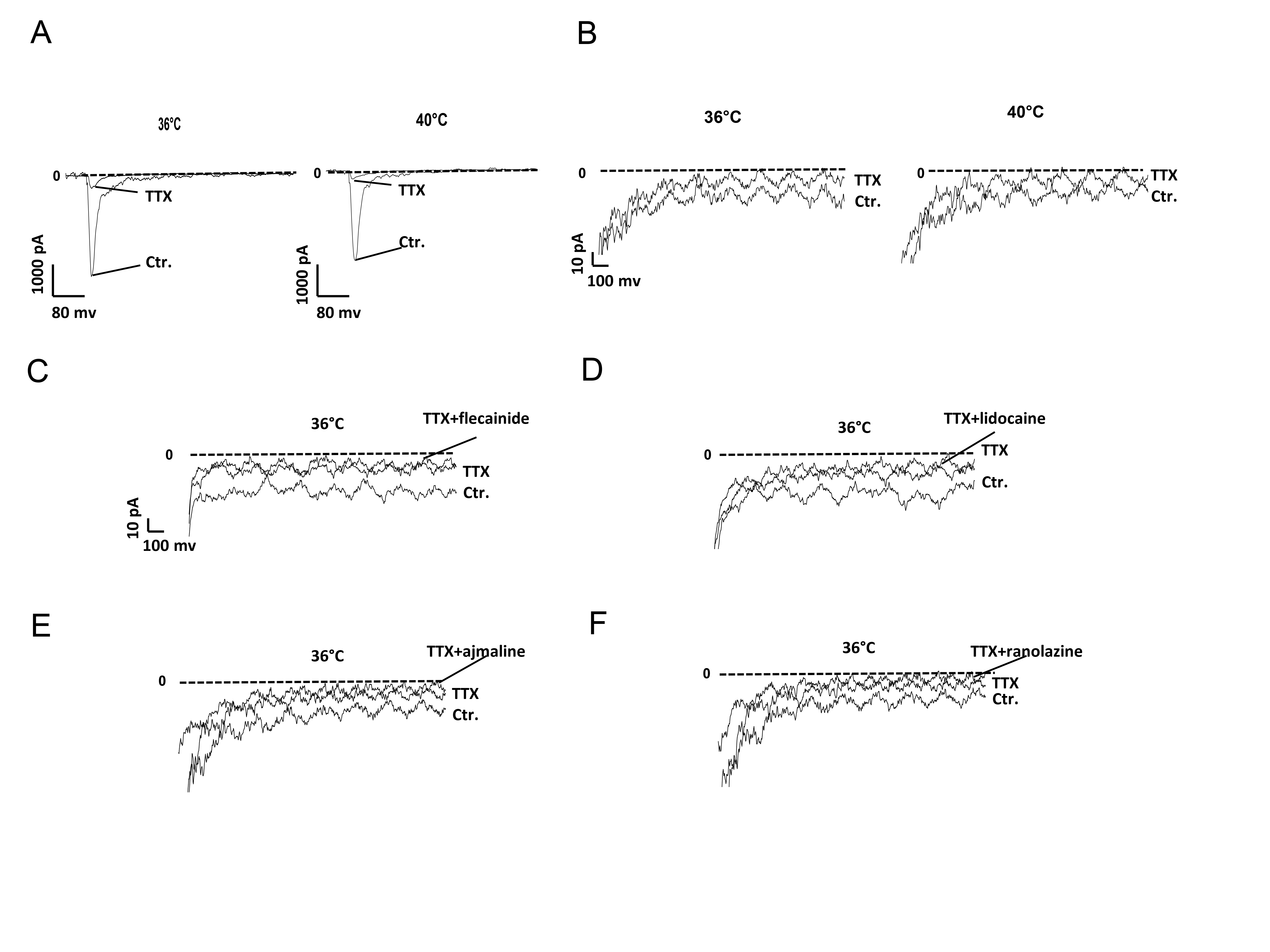

Supplement: S4 Fig — Shown are representaive INa traces recorded at -40 mV with the holding potential of -80 mV. 20 μM TTX inhibited both peak (A) and late (B) INa, and also prevented the effects of 30 μM flecainide (C), 100 μM lidocaine (D), 30 μM ajmaline (E) and 100 μM ranolazine on late INa. (TIF) [file pone.0166143.s004.tif]
