# Supplementary material for: Hyperthermia Influences the Effects of Sodium Channel Blocking Drugs in Human-Induced Pluripotent Stem Cell-Derived Cardiomyocytes
Source: PLoS One. 2016 Nov 9;11(11):e0166143. doi: 10.1371/journal.pone.0166143 (PMC5102382; doi:10.1371/journal.pone.0166143)
Supplement: S1 Table — (DOCX) [file pone.0166143.s005.docx]

**S 1 Table: Action potential properties of hiPS-CMs paced at 1Hz**

| **hiPS-CMs** | **RP APA Vmax APD50 APD90 APD90/APD50 n**  **(mV) (mV) (V/s) (ms) (ms)** |
| --- | --- |
| Ventricular | -70.3±0.7 112.9±3.3 39.1±2.1 206.2±16.5 263.0±21.3 1.3±0.02 30 |
| Atrial | -69.3±1.3 106.5±2.6 33.7±2.8 69.2±15 154.7±18.6 2.7±0.4 9 |
| Nodal | -54.5±0.5 74.5±1.5 11.0±1.0 141.0±31.0 220.0±57.0 1.5±0.1 2 |

RP, resting potential; APA, amplitude of action potential; Vmax, maximal upstroke velocity; APD50, AP duration at 50% repolarization; APD90, AP duration at 90% repolarization; APD90/APD50, ratio of APD90 to APD50; n, number of cells.
