# Supplementary material for: Hyperthermia Influences the Effects of Sodium Channel Blocking Drugs in Human-Induced Pluripotent Stem Cell-Derived Cardiomyocytes
Source: PLoS One. 2016 Nov 9;11(11):e0166143. doi: 10.1371/journal.pone.0166143 (PMC5102382; doi:10.1371/journal.pone.0166143)
Supplement: S2 Table — (DOC) [file pone.0166143.s006.doc]

|  | **S2 Table: Properties of I_Na_-kinetics in iPS-CMs at 22°C, 36°C and 40°C** | | | | | |
| --- | --- | --- | --- | --- | --- | --- |
|  | | **22°C** | **36°C** | **40°C** | **p-value**  **22°C vers. 36°C** | **p-value**  **36°C vers. 40°C** |
| Maximal peak I_Na_ (pA/pF) | | -48.2± 8.5 | -271.4±41.4 | -207.3±27.8 | **<0.05** | n.s. |
| Activation (V0.5, mV) | | -45.9±2.5 | -48.9±2.3 | -47.8±3.4 | n.s. | n.s. |
| Inactivation (V0.5, mV) | | -74.5 ±2.2 | -74.0±1.2 | -72.3±1.1 | n.s. | n.s. |
| Recovery from inactivation (Tau, ms) | | 27.8±4.8 | 15.5±1.9 | 16.2±2.2 | **<0.05** | n.s. |
|  | The values are in mean±SEM | | | | | |
